# Early SNS-Based Monitoring System for the COVID-19 Outbreak in Japan: A Population-Level Observational Study

**DOI:** 10.2188/jea.JE20200150

**Published:** 2020-08-05

**Authors:** Daisuke Yoneoka, Takayuki Kawashima, Yuta Tanoue, Shuhei Nomura, Keisuke Ejima, Shoi Shi, Akifumi Eguchi, Toshibumi Taniguchi, Haruka Sakamoto, Hiroyuki Kunishima, Stuart Gilmour, Hiroshi Nishiura, Hiroaki Miyata

**Affiliations:** 1 Department of Health Policy and Management, School of Medicine, Keio University, Tokyo, Japan; 2 Graduate School of Public Health, St. Luke’s International University, Tokyo, Japan; 3 Department of Global Health Policy, Graduate School of Medicine, The University of Tokyo, Tokyo, Japan; 4 Department of Mathematical and Computing Science, Tokyo Institute of Technology, Tokyo, Japan; 5 Institute for Business and Finance, Waseda University, Tokyo, Japan; 6 Department of Epidemiology and Biostatistics, Indiana University School of Public Health-Bloomington, Bloomington, Indiana, USA; 7 Department of Systems Pharmacology, Graduate School of Medicine, The University of Tokyo, Tokyo, Japan; 8 Laboratory for Synthetic Biology, RIKEN Center for Biosystems Dynamics Research, Osaka, Japan; 9 Department of Sustainable Health Science, Center for Preventive Medical Sciences, Chiba University, Chiba, Japan; 10 Department of Infectious Diseases, Chiba University, Chiba, Japan; 11 Department of Infectious Diseases, St. Marianna University, Kanagawa, Japan; 12 Graduate School of Medicine, Hokkaido University, Hokkaido, Japan

**Keywords:** COVID-19, Japan, large-scale monitoring system

## Abstract

**Background:**

The World Health Organization declared the novel coronavirus outbreak (COVID-19) to be a pandemic on March 11, 2020. Large-scale monitoring for capturing the current epidemiological situation of COVID-19 in Japan would improve preparation for and prevention of a massive outbreak.

**Methods:**

A chatbot-based healthcare system named COOPERA (COvid-19: Operation for Personalized Empowerment to Render smart prevention And care seeking) was developed using the LINE app to evaluate the current Japanese epidemiological situation. LINE users could participate in the system either though a QR code page in the prefectures’ websites or a banner at the top of the LINE app screen. COOPERA asked participants questions regarding personal information, preventive actions, and non-specific symptoms related to COVID-19 and their duration. We calculated daily cross correlation functions between the reported number of infected cases confirmed using polymerase chain reaction and the symptom-positive group captured by COOPERA.

**Results:**

We analyzed 206,218 participants from three prefectures reported between March 5 and 30, 2020. The mean age of participants was 44.2 (standard deviation, 13.2) years. No symptoms were reported by 96.93% of participants, but there was a significantly positive correlation between the reported number of COVID-19 cases and self-reported fevers, suggesting that massive monitoring of fever might help to estimate the scale of the COVID-19 epidemic in real time.

**Conclusions:**

COOPERA is the first real-time system being used to monitor trends in COVID-19 in Japan and provides useful insights to assist political decisions to tackle the epidemic.

## INTRODUCTION

The 2019 coronavirus disease (COVID-19) outbreak was first reported in Wuhan City, Hubei Province, China in late December 2019.^1^^,^^2^ Since then, the causative virus, severe acute respiratory syndrome coronavirus 2 (SARS-CoV-2) has spread rapidly throughout China and to 184 countries and territories; as of April 7, 2020, a total of 1,430,141 confirmed cases and 82,119 deaths have been reported worldwide.^3^^,^^4^

In Japan, there were 2,586 polymerase chain reaction (PCR)-confirmed cases with symptoms and 80 deaths as of April 7, which is relatively low compared with other countries.^5^ Clearer understanding of the current epidemiological situation of COVID-19 in Japan would improve preparation for and prevention of a massive outbreak, but the situation remains unclear due to the limited number of PCR tests conducted.^6^^,^^7^ In particular, untraceable cases have been increasing in larger cities, such as Tokyo,^5^ which suggests a shift from sporadic traceable transmission to an exponentially growing major outbreak. Unfortunately, surveillance using PCR tests or serological surveillance is not feasible because of limited resources,^8^ and because testing of mild cases often requires them to use public transport to clinics, endangering the health of others. Such surveillance would also take a long time and is not capable of responding to outbreaks in a timely manner. Therefore, rapid and large-scale monitoring for capturing the current epidemiological situation of COVID-19 in Japan is needed.^9^

On March 5, 2020, in Japan, Kanagawa prefectural government entered a collaboration with LINE Corporation,^10^ the provider of one of Japan’s largest mobile messenger applications with a claim to 83 million monthly active users, accounting for 65% of Japan’s total population. They launched a health care support system to support monitoring and follow-up of high-risk groups and potential cases of COVID-19, as well as to provide efficient support for those with mild symptoms.^11^ This system was named COvid-19: Operation for Personalized Empowerment to Render smart prevention And care seeking (COOPERA). COOPERA is also intended to support the prefectural government to rapidly grasp the epidemiological situation in the region by analyzing the data provided by the users. Kanagawa Prefecture plans to collaborate with other prefectures to develop COOPERA in a wide range of areas in Japan. The timing of the openings varied by prefecture, with the next COOPERA launch occurring on March 17 in Aichi Prefecture and March 18 in Shiga Prefecture.

In this paper, we describe the novel health care support system, COOPERA, and the results of analyses of data collected in Kanagawa, Aichi, and Shiga Prefectures by March 30, 2020. To validate whether the system captures the COVID-19 situation, we compared the data collected by COOPERA with the confirmed cases of COVID-19 reported in each prefecture.

## METHODS

### The COOPERA system

COOPERA uses a chatbot asking the participants to provide basic information, such as their current physical condition (non-specific symptoms, such as fatigue and fever) and their residence. Based on the information provided by the user, COOPERA has three major objectives:

(1) Providing individualized support for self-care and suggesting preventive behavior to avoid infection events.
  COOPERA supports better health behavior of the participants through chatbots based on the input data. The system returns individualized information related to self-care or consultation with health care providers, and further suggests preventive actions to avoid infections (eg, washing hands).
(2) Real-time follow-up and feedback to participants.
  COOPERA follows up the participants asking their health condition every other day. Depending on the user’s answers about their physical condition, age, and medical conditions, COOPERA will provide the user with information that will help them take appropriate action. For example, a fever that lasts more than 4 days, or a strong feeling of weariness (fatigue) or shortness of breath, is one of the guideposts for contacting the Coronavirus Consultation Center as defined by the Ministry of Health, Labour and Welfare. In accordance with this guideline, COOPERA also provides respondents with information on the need to contact the Coronavirus Consultation Center, as well as contact information for the center depending on where the respondent lives.
(3) Capturing the epidemiological situation to assist public health action.
  Information collected and analyzed is shared with the public health sector, which helps them implement effective measures at local to national levels.

### Participants

COOPERA launched its service in Kanagawa Prefecture on March 5, followed by Aichi Prefecture on March 17 and Shiga Prefecture on March 18. LINE users could participate in the system either though the QR code page in the prefectures’ websites or the banner at the top of the LINE app screen leading the users to the QR code page. The banner was displayed on the app users’ phones three times in Kanagawa (from 6:30 p.m. to 11:59 p.m. on March 5, 3:30 p.m. to 11:59 p.m. on March 6, and from 7:00 a.m. on March 12 to 6:59 a.m. on March 13), twice in Aichi (from 11:00 am on March 19 until 10:59 am on March 20), and twice in Shiga (from 11:00 am on March 19 until 10:59 am on March 20). Due to the company policy of LINE Corporation, the users (and the COOPERA participants) are 13 years old or older. For those who had multiple answers within 1 day, only the first answer was extracted.

### Prefectural information

Maps of the three prefectures are shown in eFigure 1. Populations of Kanagawa, Aichi, and Shiga are 9.2 million, 7.5 million, and 1.4 million, respectively, as of March 2020. In addition to the data collected through COOPERA, we extracted the number of COVID-19 cases (confirmed using PCR test) reported by each prefecture.^12^^–^^14^ As of March 30, 2020, the three prefectures had identified 130, 169, and 6 COVID-19 cases, respectively. As an overview of the health care resources of each prefecture, as of 2018, the number of hospitals per 100,000 population was 3.7, 4.3, and 4.0, respectively. The number of doctors per 100,000 population was 50.7/158.7, 45.8/161.9, and 42.9/178.0 for women/men, respectively.

### Questionnaire

COOPERA asks age, gender, occupation, medical history (malignant tumor with anticancer drugs, malignant tumor without anticancer drugs, cardiovascular diseases, kidney diseases, diabetes mellitus, receiving dialysis treatment, chronic obstructive pulmonary disease (COPD), treatment with immunosuppressants, and pregnant), preventive behaviors, residence information (zip code), and onset date of current and past month’s symptoms that are surrogate indicators of COVID-19 infection but are non-specific (presence or absence of fever, strong feeling of weariness [fatigue], or shortness of breath) and duration of these symptoms. For those who report symptoms, COOPERA asks additional questions about medical visits and clinical diagnoses at that time. In the follow-up questions, COOPERA asks their health condition again. In this study, four categories of symptoms are asked: fever above 37.5°C (Condition a), strong feeling of weariness or shortness of breath (Condition b), both Condition a and b (Condition c), or either Condition a or b (Condition d). In this study, we analyze and report the initial response to the questionnaire, and follow-up data were not accounted for. In other words, cases that developed symptoms after the initial response were not included in this analysis.

### Percentage of people with symptoms

In this study, unless otherwise noted, the percentage of condition a–d uses both current and past-month symptom responses; if on March 20, a person responds “fever above 37.5°C” for current symptoms, that person’s response is reflected in both the denominator and numerator of the March 20 percentage. Also, for the past month, if the person had a “fever above 37.5°C” on March 15, his or her answer will be reflected in the denominator for the past month, as well as in the numerator for the percentage on March 15.

### Statistical analysis

Baseline data were reported as mean (standard deviation [SD]) or proportion. For change point detection in the proportion of daily reported cases (for each Condition), a piecewise linear regression model was fitted with (at most) ten change points.^15^ The difference in slopes before and after the estimated change point(s) was tested using the Davies test.^16^^,^^17^

The relationship between two time-series data, T1) the number of cases confirmed with PCR test reported each day and T2) the proportion of the participants with any symptom onset, was examined in Kanagawa Prefecture using the sample cross correlation function (CCF) to validate that the system is capturing the epidemiological situation.^16^ Given the delay between symptom onset to confirmation observed elsewhere,^2^ we expected that T1 and T2 should be correlated with some time lag. We shifted T1 by *n* days (−10 < *n* < 10) and calculated the correlation with T2. As a sensitivity check, CCF between T2 and the weekly number of influenza cases in Kanagawa Prefecture during the study period was calculated with lag of −5 to 5 days.^18^ Statistical analyses were conducted with R software (version 3.6.0; R Foundation for Statistical Computing, Vienna, Austria). The type I error rate was fixed at 0.05.

### Ethics statement

Ethical approval was granted by the ethics committee of Keio University School of Medicine, under authorization number 20190338. We only obtained data from those who have given consent for the prefecture that administers the questionnaire to provide their response data to a third party for research use. Respondents must give their consent on the LINE chatbot before they proceed to the questionnaire response page.

## RESULTS

A total of 206,218 participants, including 124,766 (60.5%), 66,558 (32.3%), and 14,894 (7.2%) in Kanagawa, Aichi, and Shiga Prefectures, respectively, were reported from March 5 to 30, 2020. Table 1 shows the basic characteristics of the participants at the initial response date for the three prefectures combined (see eTable 1 for the prefecture-specific data). Most participants (96.93%) did not have any symptoms when enrolled in the system (ie, No-symptom group). The distribution of symptomatic conditions was 1.37%, 2.38%, 0.68%, and 3.07% for Condition a, b, c, and d, respectively. Mean and SD of age at the baseline was 44.2 (SD, 13.2) years. The age distribution of the group without symptoms was right skewed with mean age of 44.36 (SD, 13.18) years, while mean age of the group with any symptoms (ie, Condition a to d) was 38.1 (SD, 13.2) years. More women participated: 68.5% of the sample were female, 31.3% were male, and 0.2% other. The popular preventive actions were covering mouth and nose (eg, with masks or handkerchiefs) when coughing or sneezing (90.4%), washing hands with soap (89.8%), and hand disinfection with alcohol (66.4%) in the group without symptoms. Table 2 shows the proportion of participants with each preventive action stratified by symptomatic conditions on two or three time points in each prefecture, when the banners were presented and massive flow of participation was observed, showing that the preventive actions have not largely changed during the study period.

**Table 1.** Demographic characteristics of the participants by health states during the study period for the three prefectures combined, as of March 30, 2020

Total, *n* = 206,218					

	No symptom (*n* = 199,891, 96.93%)	(a) Fever ≥37.5°C (*n* = 2,821, 1.37%)	(b) Strong feeling of weariness or shortness of breath (*n* = 4,916, 2.38%)	Both (a) and (b) (*n* = 1,410, 0.68%)	Either (a) or (b) (*n* = 6,327, 3.07%)
Age, years					
Mean (SD)	44.36 (13.18)	37.67 (14.49)	38.17 (12.62)	37.44 (13.96)	38.11 (13.19)
Range (Min–Max)	13–101	13–93	13–93	13–93	13–93
13–19	6,956 (3.48, 94.96)	220 (7.80, 3.00)	239 (4.86, 3.26)	90 (6.38, 1.23)	369 (5.83, 5.04)
20–29	19,092 (9.55, 93.48)	642 (22.76, 3.14)	1,032 (20.99, 5.05)	343 (24.33, 1.68)	1,331 (21.04, 6.52)
30–39	42,844 (21.43, 95.78)	821 (29.10, 1.84)	1,485 (30.21, 3.32)	416 (29.50, 0.93)	1,890 (29.87, 4.22)
40–49	61,119 (30.58, 97.49)	605 (21.45, 0.97)	1,279 (26.02, 2.04)	311 (22.06, 0.50)	1,573 (24.86, 2.51)
50–59	45,246 (22.64, 98.29)	298 (10.56, 0.65)	632 (12.86, 1.37)	145 (10.28, 0.32)	785 (12.41, 1.71)
60–69	18,355 (9.18, 98.80)	120 (4.25, 0.65)	152 (3.09, 0.82)	50 (3.55, 0.27)	222 (3.51, 1.20)
70–79	5,654 (2.83, 97.85)	96 (3.40, 1.66)	77 (1.57, 1.33)	49 (3.48, 0.85)	124 (1.96, 2.15)
80–89	604 (0.30, 95.12)	NA	NA	NA	NA
≥90	21 (0.01, 91.30)	NA	NA	NA	NA
Sex					
Female	137,286 (68.68, 97.18)	1,648 (58.42, 1.17)	3,117 (63.41, 2.21)	788 (55.89, 0.56)	3,977 (62.86, 2.82)
Male	62,297 (31.17, 96.41)	NA	1,776 (36.13, 2.75)	NA	2,323 (36.72, 3.59)
Other	308 (0.15, 91.94)	NA	23 (0.47, 6.87)	NA	27 (0.43, 8.06)
Pregnant	2,644 (1.32, 96.81)	27 (0.96, 0.99)	78 (1.59, 2.86)	18 (1.28, 0.66)	87 (1.38, 3.19)
Occupation					
Self-employed	15,329 (7.67, 97.16)	205 (7.27, 1.30)	355 (7.22, 2.25)	112 (7.94, 0.71)	448 (7.08, 2.84)
Employees	76,616 (38.33, 96.55)	1,213 (43.00, 1.53)	2,132 (43.37, 2.69)	610 (43.26, 0.77)	2,735 (43.23, 3.45)
Public officials	10,227 (5.12, 96.92)	157 (5.57, 1.49)	259 (5.27, 2.45)	91 (6.45, 0.86)	325 (5.14, 3.08)
Student	10,633 (5.32, 95.00)	309 (10.95, 2.76)	381 (7.75, 3.40)	130 (9.22, 1.16)	560 (8.85, 5.00)
Part-time workers	39,235 (19.63, 97.85)	345 (12.23, 0.86)	680 (13.83, 1.70)	163 (11.56, 0.41)	862 (13.62, 2.15)
Unemployed	33,286 (16.65, 97.37)	374 (13.26, 1.09)	704 (14.32, 2.06)	179 (12.70, 0.52)	899 (14.21, 2.63)
Others	14,565 (7.29, 96.69)	218 (7.73, 1.45)	405 (8.24, 2.69)	125 (8.87, 0.83)	498 (7.87, 3.31)
Taking antifebrile medications (Loxonin, Caronal, etc)					
Current	4,852 (2.43, 77.53)	847 (30.02, 13.53)	1,033 (21.01, 16.51)	474 (33.62, 7.57)	1,406 (22.22, 22.47)
Past one month	11,213 (5.61, 92.74)	323 (11.45, 2.67)	743 (15.11, 6.15)	188 (13.33, 1.55)	878 (13.88, 7.26)
Diseases currently undergoing treatment (multiple answers)					
Malignant tumor with anticancer drugs	1,100 (0.55, 95.16)	33 (1.17, 2.85)	43 (0.87, 3.72)	20 (1.42, 1.73)	56 (0.89, 4.84)
Malignant tumor without anticancer drugs	2,197 (1.10, 97.26)	26 (0.92, 1.15)	49 (1.00, 2.17)	13 (0.92, 0.58)	62 (0.98, 2.74)
Cardiovascular diseases	4,112 (2.06, 95.92)	67 (2.38, 1.56)	150 (3.05, 3.50)	42 (2.98, 0.98)	175 (2.77, 4.08)
Kidney diseases	1,553 (0.78, 94.29)	37 (1.31, 2.25)	85 (1.73, 5.16)	28 (1.99, 1.70)	94 (1.49, 5.71)
Diabetes mellitus	7,152 (3.58, 96.25)	141 (5.00, 1.90)	222 (4.52, 2.99)	84 (5.96, 1.13)	279 (4.41, 3.75)
In dialysis treatment	202 (0.10, 95.28)	6 (0.21, 2.83)	10 (0.20, 4.72)	6 (0.43, 2.83)	10 (0.16, 4.72)
Chronic obstructive pulmonary disease	580 (0.29, 92.06)	18 (0.64, 2.86)	46 (0.94, 7.30)	14 (0.99, 2.22)	50 (0.79, 7.94)
Treatment with immunosuppressant	2,191 (1.10, 95.26)	52 (1.84, 2.26)	85 (1.73, 3.70)	28 (1.99, 1.22)	109 (1.72, 4.74)
Preventive measures (multiple answers)					
Washing hands in running water	115,017 (57.54, 96.88)	1,601 (56.75, 1.35)	2,882 (58.62, 2.43)	779 (55.25, 0.66)	3,704 (58.54, 3.12)
Washing hands with soap and water	179,379 (89.74, 97.11)	2,344 (83.09, 1.27)	4,146 (84.34, 2.24)	1,153 (81.77, 0.62)	5,337 (84.35, 2.89)
Hand disinfection with alcohol	132,812 (66.44, 97.20)	1,656 (58.70, 1.21)	2,969 (60.39, 2.17)	793 (56.24, 0.58)	3,832 (60.57, 2.80)
Etiquette (masks, handkerchiefs, etc) ​ in case of coughing or sneezing	180,638 (90.37, 97.06)	2,378 (84.30, 1.28)	4,265 (86.76, 2.29)	1,169 (82.91, 0.63)	5,474 (86.52, 2.94)
Take time off from school or work when ​ you have a fever or other symptoms	87,909 (43.98, 96.84)	1,480 (52.46, 1.63)	2,106 (42.84, 2.32)	715 (50.71, 0.79)	2,871 (45.38, 3.16)
Gargling with water	114,613 (57.34, 97.25)	1,406 (49.84, 1.19)	2,489 (50.63, 2.11)	651 (46.17, 0.55)	3,244 (51.27, 2.75)
Gargling with Isozine	28,792 (14.40, 96.97)	364 (12.90, 1.23)	709 (14.42, 2.39)	174 (12.34, 0.59)	899 (14.21, 3.03)
Regular ventilation	99,838 (49.95, 97.48)	1,102 (39.06, 1.08)	2,033 (41.35, 1.98)	549 (38.94, 0.54)	2,586 (40.87, 2.52)
Maintaining humidity	61,086 (30.56, 97.60)	647 (22.94, 1.03)	1,164 (23.68, 1.86)	310 (21.99, 0.50)	1,501 (23.72, 2.40)
A well-balanced diet	99,222 (49.64, 97.98)	873 (30.95, 0.86)	1,569 (31.92, 1.55)	396 (28.09, 0.39)	2,046 (32.34, 2.02)
Regular exercise	52,757 (26.39, 98.26)	420 (14.89, 0.78)	704 (14.32, 1.31)	188 (13.33, 0.35)	936 (14.79, 1.74)
Getting plenty of rest	101,439 (50.75, 97.81)	994 (35.24, 0.96)	1,750 (35.60, 1.69)	471 (33.40, 0.45)	2,273 (35.93, 2.19)
Telework	14,572 (7.29, 96.98)	169 (5.99, 1.12)	382 (7.77, 2.54)	97 (6.88, 0.65)	454 (7.18, 3.02)
Staggered commuting	19,046 (9.53, 96.85)	244 (8.65, 1.24)	496 (10.09, 2.52)	121 (8.58, 0.62)	619 (9.78, 3.15)
Avoidance of crowds other than staggered ​ commuting	49,466 (24.75, 97.76)	431 (15.28, 0.85)	893 (18.17, 1.76)	193 (13.69, 0.38)	1,131 (17.88, 2.24)
Staying up-to-date on COVID-19	119,088 (59.58, 97.46)	1,216 (43.11, 1.00)	2,451 (49.86, 2.01)	566 (40.14, 0.46)	3,101 (49.01, 2.54)
Other preventive measures	3,000 (1.50, 97.09)	22 (0.78, 0.71)	83 (1.69, 2.69)	15 (1.06, 0.49)	90 (1.42, 2.91)
No preventive measures	716 (0.36, 90.40)	42 (1.49, 5.30)	64 (1.30, 8.08)	30 (2.13, 3.79)	76 (1.20, 9.60)

SD, standard deviation.

^*^For the purpose of anonymization, all cells with less than five people are indicated as NA. In addition, when the number of persons of cells less than five can be uniquely calculated by a combination of numbers (*n*, %) of a plurality of cells, all the cells of the combination are set to NA (cells with fewer samples are labeled NA preferentially).

**Table 2.** Trends in proportions of preventive measures taken for Kanagawa, Aichi, and Shiga Prefectures

Kanagawa	Mar 6 (*n* = 23,944)	Mar 12 (*n* = 8,033)	Mar 27 (*n* = 23,625)
	
Washing hands in running water	57.73	56.67	55.38
Washing hands with soap and water	91.10	90.64	91.89
Hand disinfection with alcohol	66.28	66.36	66.23
Etiquette (masks, handkerchiefs, etc) in case of coughing or sneezing	91.68	91.39	91.18
Take time off from school or work when you have a fever or other symptoms	45.29	46.62	45.17
Gargling with water	60.67	58.42	59.64
Gargling with Isozine	16.13	15.75	14.85
Regular ventilation	48.52	48.56	54.12
Maintaining humidity	35.14	33.52	30.30
A well-balanced diet	51.77	51.33	52.16
Regular exercise	26.47	26.99	28.11
Getting plenty of rest	53.22	50.64	51.92
Telework	7.08	9.32	9.36
Staggered commuting	11.00	12.88	11.39
Avoidance of crowds other than staggered commuting	27.38	28.22	25.60
Staying up-to-date on COVID-19	59.48	61.89	61.50
Other preventive measures	1.85	1.53	1.64
No preventive measures	0.36	0.22	0.36

Aichi		Mar 19 (*n* = 22,361)	Mar 27 (*n* = 16,083)
	
Washing hands in running water		59.13	59.39
Washing hands with soap and water		85.90	86.28
Hand disinfection with alcohol		65.05	64.53
Etiquette (masks, handkerchiefs, etc) in case of coughing or sneezing		89.09	88.67
Take time off from school or work when you have a fever or other symptoms		40.92	40.75
Gargling with water		52.49	54.26
Gargling with Isozine		13.59	12.34
Regular ventilation		47.98	47.21
Maintaining humidity		28.06	27.51
A well-balanced diet		45.82	46.62
Regular exercise		25.39	25.06
Getting plenty of rest		48.09	50.07
Telework		3.83	4.28
Staggered commuting		5.64	5.88
Avoidance of crowds other than staggered commuting		21.87	21.78
Staying up-to-date on COVID-19		54.54	58.15
Other preventive measures		1.40	1.66
No preventive measures		0.51	0.52

Shiga		Mar 19 (*n* = 6,522)	Mar 27 (*n* = 3,283)
	
Washing hands in running water		55.52	55.47
Washing hands with soap and water		86.55	86.45
Hand disinfection with alcohol		64.21	62.93
Etiquette (masks, handkerchiefs, etc) in case of coughing or sneezing		87.47	88.33
Take time off from school or work when you have a fever or other symptoms		40.34	38.99
Gargling with water		51.30	51.51
Gargling with Isozine		13.43	12.00
Regular ventilation		43.65	43.74
Maintaining humidity		26.76	23.73
A well-balanced diet		45.31	47.58
Regular exercise		25.50	25.65
Getting plenty of rest		46.20	48.04
Telework		2.70	2.95
Staggered commuting		3.74	3.75
Avoidance of crowds other than staggered commuting		19.23	18.58
Staying up-to-date on COVID-19		55.57	57.08
Other preventive measures		1.26	1.22
No preventive measures		0.51	0.61

**Figure 1.**
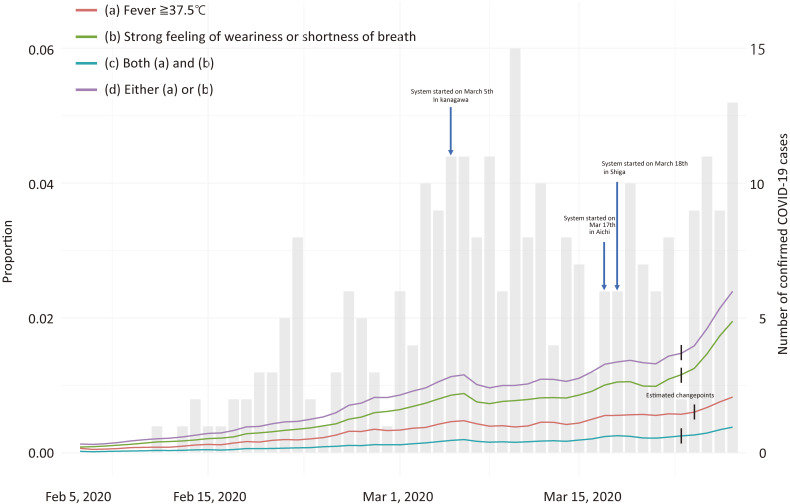
Trend in the proportion of non-specific symptoms in three prefectures. Red line, green line, blue line, and purple line indicate the proportion of participants who have a fever (a), a strong feeling of weariness or shortness of breath (b), both (a) and (b), and either (a) or (b), respectively. Gray bars indicate the reported number of COVID-19-positive cases in three prefectures.

**Figure 2.**
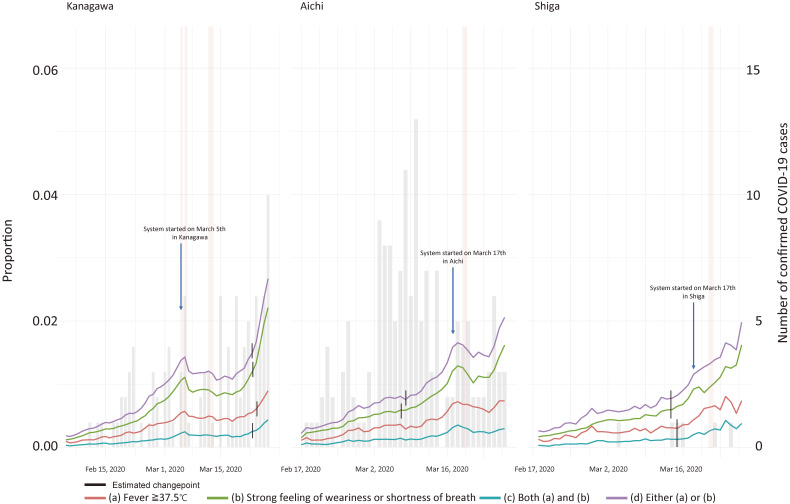
Monthly trend in each prefecture (left: Kanagawa, middle: Aichi, right: Shiga). Red line, green line, blue line, and purple line indicate the proportion of participants who have a fever (a), a strong feeling of weariness or shortness of breath (b), both (a) and (b), and either (a) or (b), respectively. Gray bar indicates the reported number of COVID-19-positive cases in each prefecture. Pink bands are the banner periods.

The timeline of the proportion of each condition for each day from February 1 to March 30, 2020, is shown in Figure 1 and Figure 2. Figure 1 depicts the proportions and the number of confirmed cases in the three prefectures. The significant change points were observed on March 24, 2020 for Condition a (*P* = 0.036), and March 23, 2020 for Condition b, c and d (*P* = 0.012, *P* < 0.001, and *P* = 0.024, respectively). Figure 2 indicates the proportions for each prefecture, also showing that the significant change-points were March 24, 2020 for Condition a (*P* < 0.001) and March 23, 2020 for Condition b, c and d (all *P* < 0.001) in Kanagawa Prefecture; March 7, 2020 for Condition b (*P* < 0.001) and March 8, 2020 for Condition d (*P* < 0.001) in Aichi Prefecture; and March 14, 2020 for Condition b and d (both *P* < 0.001), and March 15, 2020 for Condition c (*P* = 0.039) in Shiga Prefecture.

**Figure 3.**
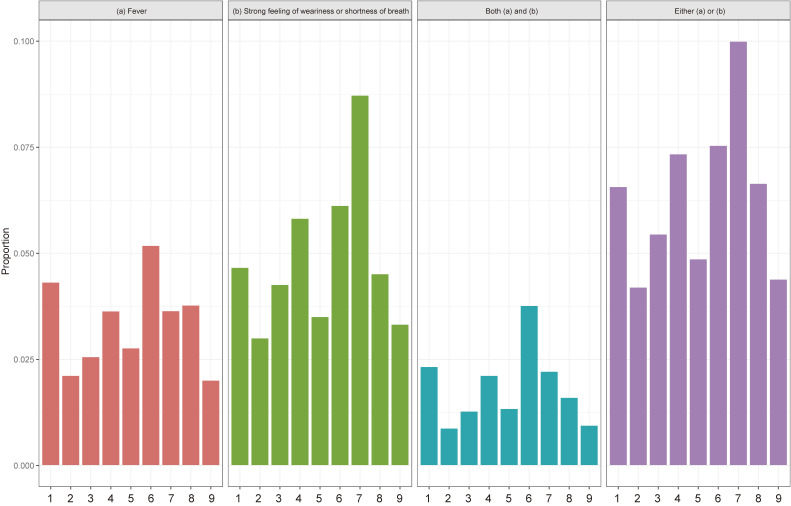
The proportion of participants with each non-specific symptom among participants with comorbidities and pregnant. 1. Malignant tumor with anticancer drugs, 2. Malignant tumor without anticancer drugs, 3. Cardiovascular diseases, 4. Kidney diseases, 5. Diabetes mellitus, 6. In dialysis treatment, 7. Chronic obstructive pulmonary disease, 8. Treatment with immunosuppressant, 9. Pregnant.

The proportion of participants with each non-specific symptom among participants with comorbidities and who were pregnant are shown in Figure 3. The participants with COPD, immunosuppressant treatment, or malignant tumor with anticancer drugs had fever more than those with the other comorbidities and pregnancy.

**Figure 4.**
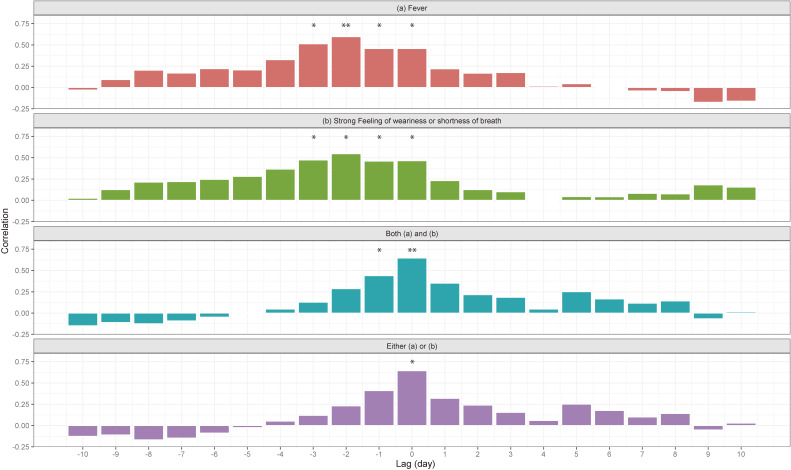
Cross correlation function between the time series of reported number of COVID-19 cases and the shifted time series of the proportion of non-specific symptoms (shifted days between −10 to 10). ^**^P < 0.01, ^*^P < 0.05

Last, we validated whether COOPERA captures the COVID-19 situation by comparing the proportions in Kanagawa Prefecture with the confirmed cases of COVID-19 (Figure 4). We found significant correlation between T1 and T2 with 0 to 3 days delay, suggesting that non-specific symptoms reported in COOPERA captures the COVID-19 epidemic in the region and is a powerful tool to infer the trend of COVID-19 pandemic. Similar results were obtained in a sensitivity analysis where the study period was divided into two periods before and after the start date. In addition, the CCF between the proportion of the participants with fever and the reported number of influenza cases in Kanagawa Prefecture was calculated (eFigure 2), showing no significant positive correlation between them (with *P* = 0.56–0.99 for each lag between −5 to 5 days).

## DISCUSSION

In this study, we used a novel health care support system, COOPERA, and analyzed over 200,000 observations in three prefectures in Japan. We found that 96.93% of participants had no symptoms, and the proportion of participants with fever significantly correlated with the number of COVID-19-positive cases after 0 to 3 days, suggesting that a questionnaire-based massive epidemiological monitoring may be able to capture the actual epidemic situation. This result was robust even when we divided the study period into two periods before and after the start date. However, the number of participants with fever includes both COVID-19 cases and other cases with fever due to common cold or influenza. Thus, it is possible that the proportions we have shown here do not directly represent COVID-19 prevalence. To address this, we calculated the CCF between the proportion and the reported number of influenza cases and obtained no significant positive correlation between them (eFigure 2). This suggests the system has (at least partially) captured the COVID-19 epidemiological situation.

Like several other infectious diseases, including SARS-CoV and MERS-CoV, SARS-CoV-2 is a coronavirus capable of human-to-human transmission, and those who have comorbidities are at high risk of mortality.^19^^–^^21^ Our results imply that those who have chronic comorbidities, especially COPD, immunosuppressant treatment, or malignant tumor, might be more susceptible to COVID-19 infection. Further, in terms of preventive action, cough etiquette and handwashing with soap were common preventive actions (around 90%), while less than 10% of the participants executed telework and stagger commuting hours during the study period. This result might indicate that most people are already implementing individual-level preventive actions, such as handwashing, while society-level preventive efforts, which require governmental or community support, are still not being properly implemented and need to be more strongly encouraged. Given that teleworking in particular is not an individual choice and is a very important non-pharmaceutical intervention, the governments of these prefectures need to consider further measures to ensure companies make teleworking available to their staff on a wider basis.

The characteristics of COOPERA can be contrasted with existing infectious disease surveillance.^22^^,^^23^ For example, there is a survey in Japan that collects information on pharmacy dispensing with the aim of early detection of domestic outbreaks of influenza. In addition, there is a survey on the implementation of school closure due to outbreaks of influenza in order to capture the epidemic. On the other hand, COOPERA is characterized by the ability to detect signs of an epidemic before it occurs in an area. With COOPERA, LINE users can immediately register their symptoms via LINE. In other words, it has a high degree of immediacy in that it can collect data before visits to pharmacies, school closures, and other measures are taken. In addition, COOPERA can also track the change over time of a user’s individual symptoms (eg, when they appeared and how many days until they recovered).

Because personal protective equipment is in short supply and there is a risk of infection among healthcare professionals, it is not realistic to increase the number of PCR tests immediately. The fact that there is a correlation between the number of PCR-positive cases and the proportion of symptoms detected using COOPERA in this study suggests that COOPERA may be able to detect the scale of the spread of infection and the location of the outbreak when the number of infected cases increases and it becomes even more difficult to grasp the actual situation by PCR.

This study has several limitations. The first one is bias of participants. Since COOPERA monitoring was based on the online platform LINE and used a non-random sampling scheme, our findings might be influenced by selection bias: those who have no access to the internet or smartphone may be underrepresented. In addition, there is a possibility that LINE users who have a relatively high level of health awareness or are worried about some symptoms tend to respond to the system. Therefore, the proportion of participants with symptoms might have overestimated the prevalence of people with symptoms. In addition, it should be noted that the results showed a low percentage of positive symptoms in the day when the banner was displayed. There is a possibility that the participants who answered the questions on the day the banner was displayed were not suffered symptoms by disease conditions preventing them from joining the system at the moment, consequently underestimating the actual proportion of people with the symptoms. Conversely, the participants who answered the questions before or after the period of banner display could be more motivated to participate in the system if any symptoms present, which might end up with overestimating the percentage of symptomatic participants. Unfortunately, we only have information on the population of LINE users, which is claimed by the company to be about 83 million in total, and there is no detailed distribution data regarding the participants’ background such as sex, age, or prefecture. Therefore, it is difficult to verify selection bias because it is not possible to evaluate the response rate among users and compare the demographic characteristics of non-respondents and respondents. In addition, since the detailed and analyzable data on those who received PCR testing was not publicly available, it should be noted that the CCF result might include this bias, which is difficult to examine.

Second, it should be noted that the system could not avoid recall bias. In this study, COOPERA monitoring included questions regarding symptoms in the last month. People may not remember the symptoms in the last month accurately. This recall bias may have created upward trend of the proportion with symptoms toward the start of the system.

Third, given the selection bias and recall bias in COOPERA, Figure 1 may give an exaggerated impression that the trend of the number of COVID-19 has been *dramatically* changed during the study period. In the figure, we showed the proportion of participants who have symptoms reflecting past symptomatic experience. However, trends in the daily proportion using only the “current symptom at each study period” data are shown in eFigure 3. No exponential trends were found, suggesting that the results of this study may overestimate the true prevalence of symptoms. However, if only “current symptoms” are used, the number of respondents varies greatly from day to day, and the calculation of the percentage is not stable, as can be observed in eFigure 3. For example, in Kanagawa Prefecture, there are several peaks, but the timing of the peak at the bottom coincides with the timing of the COOPERA information released at the Kanagawa Governor’s press conference. This peak may have occurred as a result of the increase in the number of respondents following the press conference and media coverage of COOPERA, with the percentage stabilizing only for that day. By taking into account the “symptoms of the past month”, the percentage calculation is stabilized as the information of the respondents on a given day is used to calculate the percentage over the past month (with a recall bias, of course).

### Conclusions

In summary, this study is the first report based on a large-scale (over 200,000 participants) health care support system in Japan. Significant correlation between the time trend of participants with symptom with the time trend of PCR-confirmed cases supports the utility of the system in monitoring the COVID-19 epidemiological situation in Japan.

## APPENDIX A. SUPPLEMENTARY DATA

eTable 1. Demographic characteristics of the participants by health states during the study period for Kanagawa, Aichi, and Shiga prefecture, as of March 30, 2020

eFigure 1. Map of selected three prefectures in Japan.

eFigure 2. Weekly trend in the proportion of participants who have a fever (left) and the reported number of influenza cases (middle) and cross correlation function between them (right). Blue line in right panel is standard deviation. ACF is autocorrelation function.

eFigure 3. Trend in the proportion of new participants who have non-specific symptoms in each prefecture (left: Kanagawa, middle: Aichi, right: Shiga). Red line, green line, blue line, and purple line indicate the proportion of participants who have a fever (a), a strong feeling of weariness or shortness of breath (b), both (a) and (b), and either (a) or (b), respectively.
