# Supplementary material for: Early SNS-Based Monitoring System for the COVID-19 Outbreak in Japan: A Population-Level Observational Study
Source: J Epidemiol. 2020 Aug 5;30(8):362–70. doi: 10.2188/jea.JE20200150 (PMC7348074; doi:10.2188/jea.JE20200150)
Supplement: Supplementary file 1 [file je-30-362-s001.pdf]

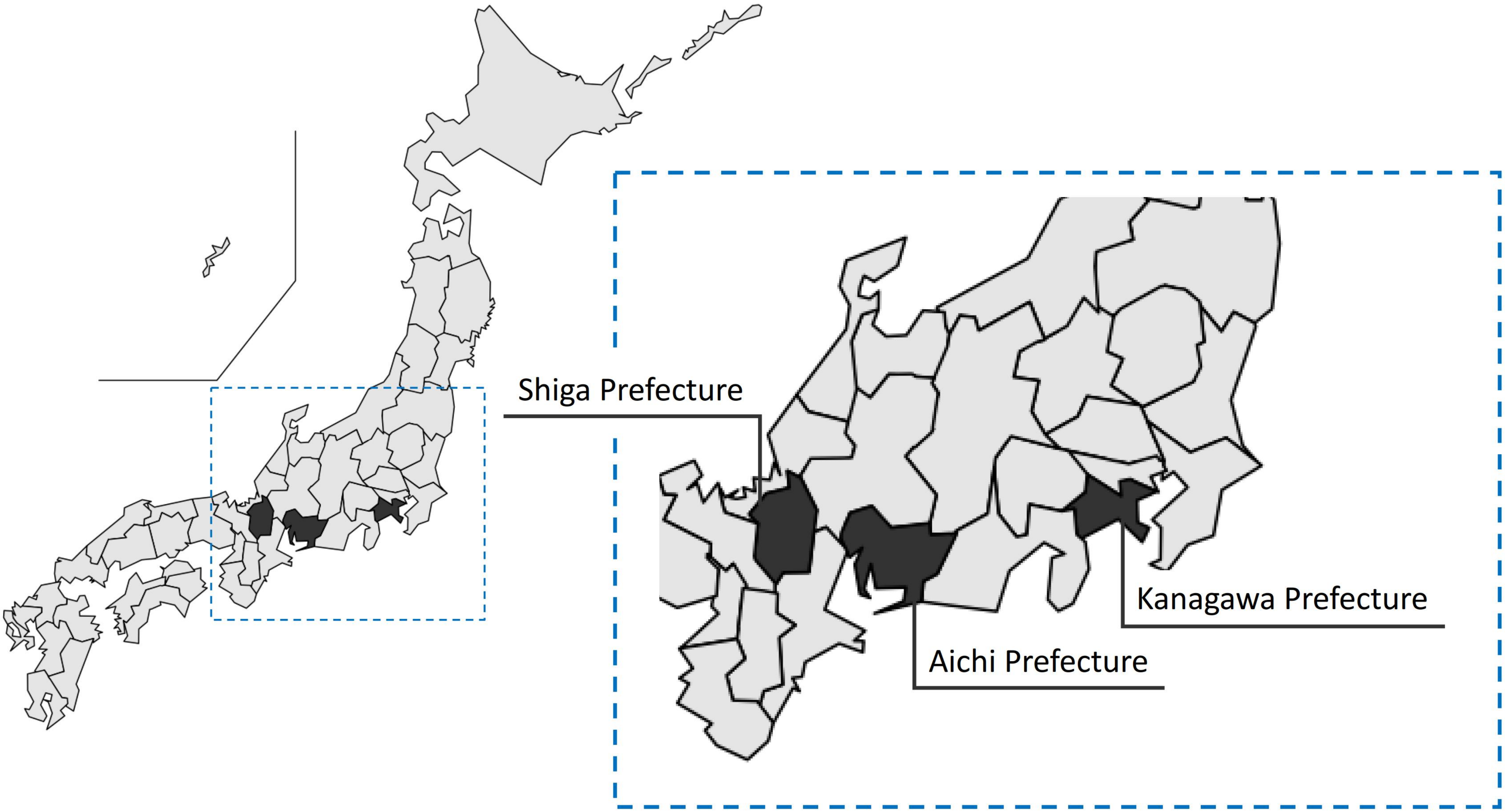

eFigure 1. Map of selected three prefectures in Japan.

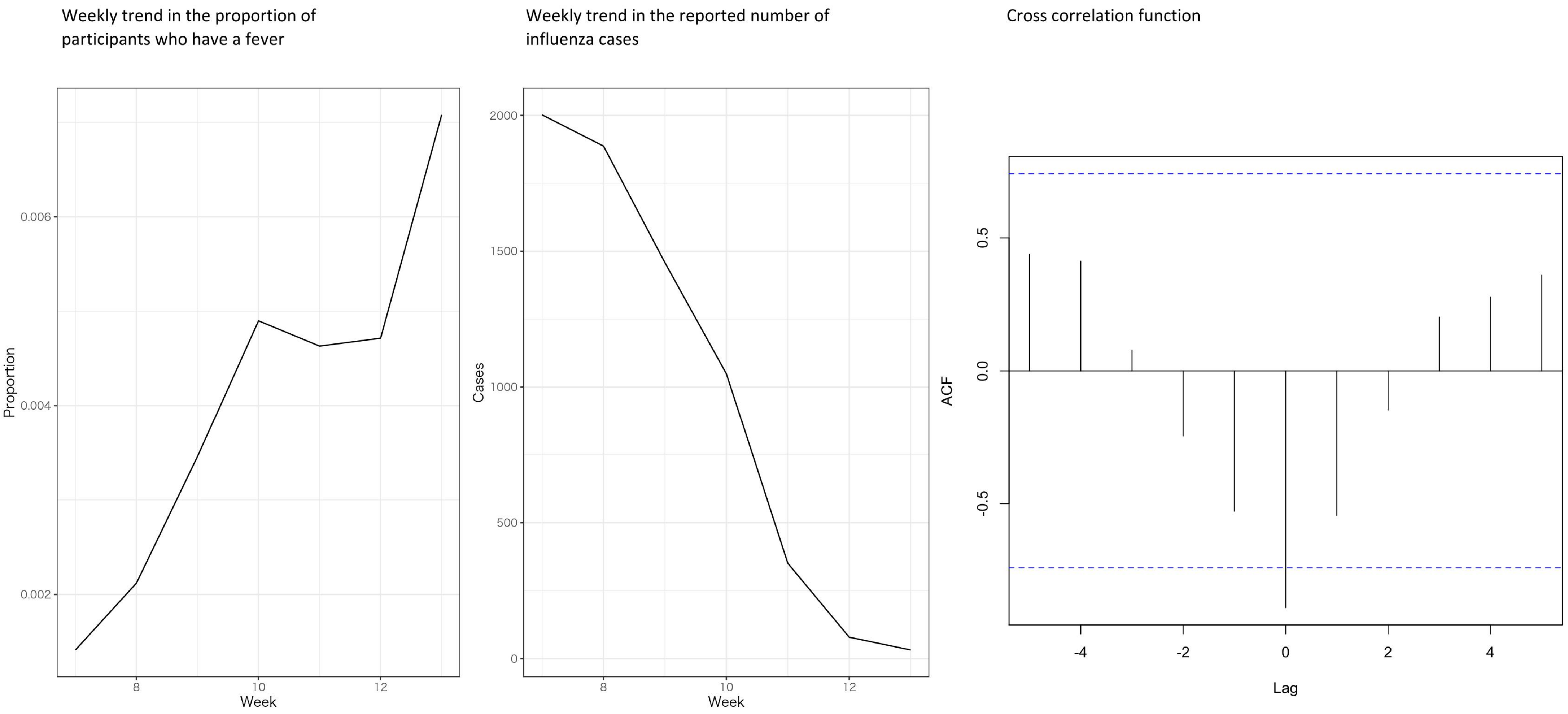

eFigure 2. Weekly trend in the proportion of participants who have a fever (left) and the reported number of influenza cases (middle) and cross correlation function between them (right). Blue line in right panel is standard deviation. ACF is autocorrelation function.

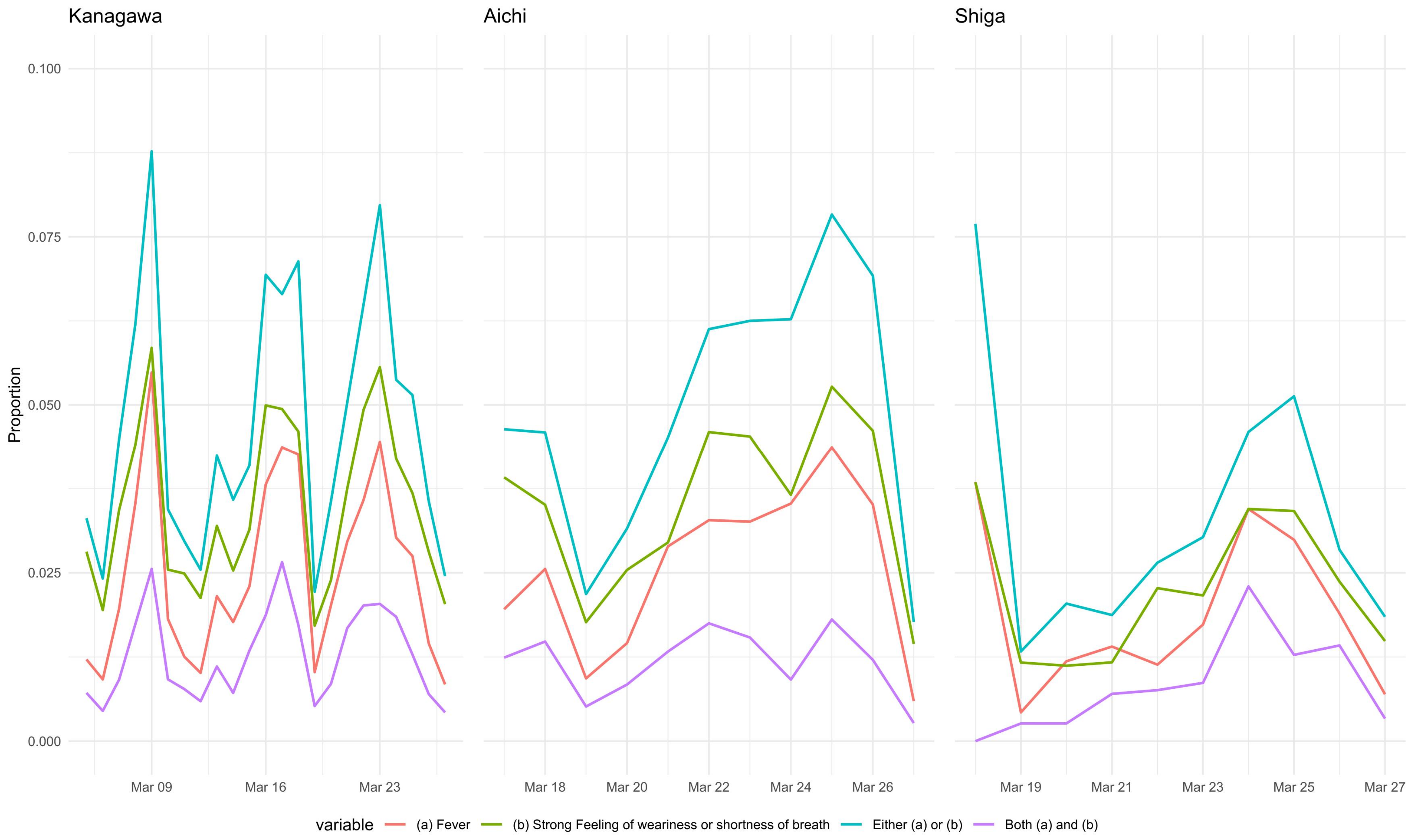

eFigure 3. Trend in the proportion of new participants who have non-specific symptoms in each prefecture (left: Kanagawa, middle: Aichi, right: Shiga). Red line, green line, blue line, and purple line indicate the proportion of participants who have a fever (a), a strong feeling of weariness or shortness of breath (b), both (a) and (b), and either (a) or (b), respectively.

eTable 1. Demographic characteristics of the participants by health states during the study period for Kanagawa, Aichi, and Shiga prefecture, as of March 30, 2020

Kanagawa, n=124,766					
	No symptom (n=120,635, 96.69%)	(a) Fever ≥37.5℃ (n=1,831, 1.47%)	(b) Strong feeling of weariness or shortness of breath (n=3,223, 2.58%)	Both (a) and (b) (n=923, 0.74%)	Either (a) or (b) (n=4,131, 3.31%)
Age, years					
Mean (SD)	44.82 (12.98)	37.57 (14.31)	38.53 (12.48)	37.6 (13.77)	38.31 (13.05)
Range (Min–Max)	13–101	13–93	13–93	13–93	13–93
13–19	3,919 (3.25, 94.39)	147 (8.03, 3.54)	144 (4.47, 3.47)	58 (6.28, 1.40)	233 (5.64, 5.61)

20–29	10,463 (8.67, 92.56)	409 (22.34, 3.62)	653 (20.26, 5.78)	221 (23.94, 1.96)	841 (20.36, 7.44)
30–39	24,610 (20.40, 95.34)	517 (28.24, 2.00)	948 (29.41, 3.67)	261 (28.28, 1.01)	1,204 (29.15, 4.66)
40–49	38,072 (31.56, 97.23)	420 (22.94, 1.07)	885 (27.46, 2.26)	220 (23.84, 0.56)	1,085 (26.26, 2.77)
50–59	28,751 (23.83, 98.16)	202 (11.03, 0.69)	437 (13.56, 1.49)	101 (10.94, 0.34)	538 (13.02, 1.84)
60–69	11,011 (9.13, 98.82)	67 (3.66, 0.60)	94 (2.92, 0.84)	30 (3.25, 0.27)	131 (3.17, 1.18)
70–79	3,397 (2.82, 97.70)	58 (3.17, 1.67)	50 (1.55, 1.44)	28 (3.03, 0.81)	80 (1.94, 2.30)

80–89	398 (0.33, 95.67)	NA	NA	NA	NA
≥90	14 (0.01, 93.33)	NA	NA	NA	NA
Sex					
Female	83,320 (69.07, 96.93)	1,074 (58.66, 1.25)	2,086 (64.72, 2.43)	524 (56.77, 0.61)	2,636 (63.81, 3.07)
Male	37,129 (30.78, 96.18)	NA	1,121 (34.78, 2.90)	NA	1,475 (35.71, 3.82)
Other	186 (0.15, 90.29)	NA	16 (0.50, 7.77)	NA	20 (0.48, 9.71)
Pregnant	1,428 (1.18, 96.95)	13 (0.71, 0.88)	42 (1.30, 2.85)	10 (1.08, 0.68)	45 (1.09, 3.05)
Occupation					

Self-employed	8,945 (7.41, 96.95)	125 (6.83, 1.35)	228 (7.07, 2.47)	72 (7.80, 0.78)	281 (6.80, 3.05)
Employees	47,564 (39.43, 96.29)	797 (43.53, 1.61)	1,434 (44.49, 2.90)	397 (43.01, 0.80)	1,834 (44.40, 3.71)
Public officials	5,442 (4.51, 96.30)	99 (5.41, 1.75)	166 (5.15, 2.94)	56 (6.07, 0.99)	209 (5.06, 3.70)
Student	6,116 (5.07, 94.53)	208 (11.36, 3.21)	234 (7.26, 3.62)	88 (9.53, 1.36)	354 (8.57, 5.47)
Part–time workers	23,040 (19.10, 97.72)	221 (12.07, 0.94)	420 (13.03, 1.78)	103 (11.16, 0.44)	538 (13.02, 2.28)
Unemployed	20,447 (16.95, 97.26)	234 (12.78, 1.11)	461 (14.30, 2.19)	120 (13.00, 0.57)	575 (13.92, 2.74)

Others	9,081 (7.53, 96.39)	147 (8.03, 1.56)	280 (8.69, 2.97)	87 (9.43, 0.92)	340 (8.23, 3.61)
Taking antifebrile medications (Loxonin, Caronal, etc.)					
Current	2,747 (2.28, 75.59)	547 (29.87, 15.05)	644 (19.98, 17.72)	304 (32.94, 8.37)	887 (21.47, 24.41)
Past one month	6,590 (5.46, 92.30)	202 (11.03, 2.83)	472 (14.64, 6.61)	124 (13.43, 1.74)	550 (13.31, 7.70)
Diseases currently undergoing treatment (multiple answers)					
Malignant tumor with anticancer drugs	624 (0.52, 94.26)	20 (1.09, 3.02)	30 (0.93, 4.53)	12 (1.30, 1.81)	38 (0.92, 5.74)

Malignant tumor without anticancer drugs	1,349 (1.12, 96.77)	NA	37 (1.15, 2.65)	NA	45 (1.09, 3.23)
Cardiovascular diseases	2,481 (2.06, 95.90)	44 (2.40, 1.70)	92 (2.85, 3.56)	30 (3.25, 1.16)	106 (2.57, 4.10)
Kidney diseases	869 (0.72, 94.15)	22 (1.20, 2.38)	50 (1.55, 5.42)	18 (1.95, 1.95)	54 (1.31, 5.85)
Diabetes mellitus	4,206 (3.49, 95.92)	85 (4.64, 1.94)	144 (4.47, 3.28)	50 (5.42, 1.14)	179 (4.33, 4.08)
In dialysis treatment	NA	NA	NA	NA	NA
Chronic obstructive pulmonary disease	NA	NA	NA	11 (1.19, 2.69)	NA
Treatment with immunosuppressant	1,339 (1.11, 95.30)	31 (1.69, 2.21)	52 (1.61, 3.70)	17 (1.84, 1.21)	66 (1.60, 4.70)

Preventive measures (multiple answers)					
Washing hands in running water	68,695 (56.94, 96.64)	1,031 (56.31, 1.45)	1,861 (57.74, 2.62)	507 (54.93, 0.71)	2,385 (57.73, 3.36)
Washing hands with soap and water	110,991 (92.01, 96.88)	1,555 (84.93, 1.36)	2,794 (86.69, 2.44)	780 (84.51, 0.68)	3,569 (86.40, 3.12)
Hand disinfection with alcohol	81,230 (67.34, 96.97)	1,092 (59.64, 1.30)	1,972 (61.19, 2.35)	527 (57.10, 0.63)	2,537 (61.41, 3.03)
Etiquette (masks, handkerchiefs, etc.) in case of coughing or sneezing	110,397 (91.51, 96.80)	1,580 (86.29, 1.39)	2,863 (88.83, 2.51)	791 (85.70, 0.69)	3,652 (88.40, 3.20)
Take time off from school or work when you have a fever or other symptoms	55,797 (46.25, 96.61)	1,002 (54.72, 1.73)	1,442 (44.74, 2.50)	484 (52.44, 0.84)	1,960 (47.45, 3.39)

Gargling with water	72,605 (60.19, 97.00)	955 (52.16, 1.28)	1,730 (53.68, 2.31)	443 (48.00, 0.59)	2,242 (54.27, 3.00)
Gargling with Isozine	18,373 (15.23, 96.78)	242 (13.22, 1.27)	492 (15.27, 2.59)	123 (13.33, 0.65)	611 (14.79, 3.22)
Regular ventilation	62,347 (51.68, 97.26)	742 (40.52, 1.16)	1,391 (43.16, 2.17)	374 (40.52, 0.58)	1,759 (42.58, 2.74)
Maintaining humidity	39,640 (32.86, 97.41)	435 (23.76, 1.07)	835 (25.91, 2.05)	214 (23.19, 0.53)	1,056 (25.56, 2.59)
A well–balanced diet	62,850 (52.10, 97.79)	617 (33.70, 0.96)	1,093 (33.91, 1.70)	292 (31.64, 0.45)	1,418 (34.33, 2.21)
Regular exercise	32,812 (27.20, 98.08)	287 (15.67, 0.86)	490 (15.20, 1.46)	136 (14.73, 0.41)	641 (15.52, 1.92)

Getting plenty of rest	63,169 (52.36, 97.60)	682 (37.25, 1.05)	1,204 (37.36, 1.86)	331 (35.86, 0.51)	1,555 (37.64, 2.40)
Telework	11,407 (9.46, 96.78)	138 (7.54, 1.17)	320 (9.93, 2.71)	78 (8.45, 0.66)	380 (9.20, 3.22)
Staggered commuting	14,726 (12.21, 96.71)	187 (10.21, 1.23)	404 (12.53, 2.65)	90 (9.75, 0.59)	501 (12.13, 3.29)
Avoidance of crowds other than staggered commuting	32,689 (27.10, 97.59)	312 (17.04, 0.93)	637 (19.76, 1.90)	141 (15.28, 0.42)	808 (19.56, 2.41)
Staying up-to-date on COVID-19	74,106 (61.43, 97.22)	836 (45.66, 1.10)	1,691 (52.47, 2.22)	411 (44.53, 0.54)	2,116 (51.22, 2.78)
Other preventive measures	1,896 (1.57, 96.78)	16 (0.87, 0.82)	58 (1.80, 2.96)	11 (1.19, 0.56)	63 (1.53, 3.22)

No preventive measures	322 (0.27, 88.71)	20 (1.09, 5.51)	34 (1.05, 9.37)	13 (1.41, 3.58)	41 (0.99, 11.29)

Aichi, n=66,558					
	No symptom (n=64,646, 97.13%)	(a) Fever ≥37.5℃ (n=866, 1.30%)	(b) Strong feeling of weariness or shortness of breath (n=1,472, 2.21%)	Both (a) and (b) (n=426, 0.64%)	Either (a) or (b) (n=1,912, 2.87%)
Age, years					
Mean (SD)	43.25 (13.33)	37.57 (14.59)	37.16 (12.49)	36.6 (13.78)	37.47 (13.2)
Range (Min–Max)	13–95	13–90	13–86	13–86	13–90
13–19	2,563 (3.96, 95.78)	63 (7.27, 2.35)	78 (5.30, 2.91)	28 (6.57, 1.05)	113 (5.91, 4.22)

20–29	7,338 (11.35, 94.27)	211 (24.36, 2.71)	348 (23.64, 4.47)	113 (26.53, 1.45)	446 (23.33, 5.73)
30–39	15,259 (23.60, 96.20)	267 (30.83, 1.68)	469 (31.86, 2.96)	133 (31.22, 0.84)	603 (31.54, 3.80)
40–49	18,768 (29.03, 97.81)	161 (18.59, 0.84)	340 (23.10, 1.77)	81 (19.01, 0.42)	420 (21.97, 2.19)
50–59	13,299 (20.57, 98.46)	82 (9.47, 0.61)	165 (11.21, 1.22)	39 (9.15, 0.29)	208 (10.88, 1.54)
60–69	5,605 (8.67, 98.66)	45 (5.20, 0.79)	47 (3.19, 0.83)	16 (3.76, 0.28)	76 (3.97, 1.34)
70–79	1,655 (2.56, 97.93)	30 (3.46, 1.78)	19 (1.29, 1.12)	14 (3.29, 0.83)	35 (1.83, 2.07)

80–89	152 (0.24, 93.83)	NA	NA	NA	NA
≥90	7 (0.01, 87.50)	NA	NA	NA	NA
Sex					
Female	44,044 (68.13, 97.43)	503 (58.08, 1.11)	890 (60.46, 1.97)	232 (54.46, 0.51)	1,161 (60.72, 2.57)
Male	20,498 (31.71, 96.50)	NA	575 (39.06, 2.71)	NA	744 (38.91, 3.50)
Other	104 (0.16, 93.69)	NA	7 (0.48, 6.31)	NA	7 (0.37, 6.31)
Pregnant	1,033 (1.60, 96.27)	13 (1.50, 1.21)	34 (2.31, 3.17)	7 (1.64, 0.65)	40 (2.09, 3.73)
Occupation					

Self-employed	5,256 (8.13, 97.28)	68 (7.85, 1.26)	112 (7.61, 2.07)	33 (7.75, 0.61)	147 (7.69, 2.72)
Employees	24,243 (37.50, 96.80)	367 (42.38, 1.47)	621 (42.19, 2.48)	187 (43.90, 0.75)	801 (41.89, 3.20)
Public officials	3,728 (5.77, 97.39)	52 (6.00, 1.36)	79 (5.37, 2.06)	31 (7.28, 0.81)	100 (5.23, 2.61)
Student	3,755 (5.81, 95.52)	89 (10.28, 2.26)	124 (8.42, 3.15)	37 (8.69, 0.94)	176 (9.21, 4.48)
Part–time workers	13,030 (20.16, 97.92)	107 (12.36, 0.80)	226 (15.35, 1.70)	56 (13.15, 0.42)	277 (14.49, 2.08)
Unemployed	10,194 (15.77, 97.37)	118 (13.63, 1.13)	206 (13.99, 1.97)	49 (11.50, 0.47)	275 (14.38, 2.63)

Others	4,440 (6.87, 97.03)	65 (7.51, 1.42)	104 (7.07, 2.27)	33 (7.75, 0.72)	136 (7.11, 2.97)
Taking antifebrile medications (Loxonin, Caronal, etc.)					
Current	1,640 (2.54, 79.19)	255 (29.45, 12.31)	320 (21.74, 15.45)	144 (33.80, 6.95)	431 (22.54, 20.81)
Past one month	3,490 (5.40, 92.84)	104 (12.01, 2.77)	218 (14.81, 5.80)	53 (12.44, 1.41)	269 (14.07, 7.16)
Diseases currently undergoing treatment (multiple answers)					
Malignant tumor with anticancer drugs	371 (0.57, 96.11)	11 (1.27, 2.85)	11 (0.75, 2.85)	7 (1.64, 1.81)	15 (0.78, 3.89)

Malignant tumor without anticancer drugs	651 (1.01, 97.89)	9 (1.04, 1.35)	NA	NA	14 (0.73, 2.11)
Cardiovascular diseases	1,268 (1.96, 95.63)	21 (2.42, 1.58)	48 (3.26, 3.62)	11 (2.58, 0.83)	58 (3.03, 4.37)
Kidney diseases	544 (0.84, 94.12)	12 (1.39, 2.08)	31 (2.11, 5.36)	9 (2.11, 1.56)	34 (1.78, 5.88)
Diabetes mellitus	2,371 (3.67, 96.62)	48 (5.54, 1.96)	62 (4.21, 2.53)	27 (6.34, 1.10)	83 (4.34, 3.38)
In dialysis treatment	NA	NA	NA	NA	NA
Chronic obstructive pulmonary disease	174 (0.27, 92.55)	NA	13 (0.88, 6.91)	NA	14 (0.73, 7.45)
Treatment with immunosuppressant	692 (1.07, 95.58)	13 (1.50, 1.80)	26 (1.77, 3.59)	7 (1.64, 0.97)	32 (1.67, 4.42)
Preventive measures (multiple answers)					

Washing hands in running water	38,166 (59.04, 97.08)	505 (58.31, 1.28)	882 (59.92, 2.24)	241 (56.57, 0.61)	1,146 (59.94, 2.92)
Washing hands with soap and water	55,733 (86.21, 97.31)	686 (79.21, 1.20)	1,179 (80.10, 2.06)	326 (76.53, 0.57)	1,539 (80.49, 2.69)
Hand disinfection with alcohol	42,230 (65.33, 97.42)	487 (56.24, 1.12)	860 (58.42, 1.98)	230 (53.99, 0.53)	1,117 (58.42, 2.58)
Etiquette (masks, handkerchiefs, etc.) in case of coughing or sneezing	57,439 (88.85, 97.30)	704 (81.29, 1.19)	1,229 (83.49, 2.08)	337 (79.11, 0.57)	1,596 (83.47, 2.70)
Take time off from school or work when you have a fever or other symptoms	26,335 (40.74, 97.06)	420 (48.50, 1.55)	582 (39.54, 2.14)	203 (47.65, 0.75)	799 (41.79, 2.94)

Gargling with water	34,514 (53.39, 97.51)	394 (45.50, 1.11)	671 (45.58, 1.90)	183 (42.96, 0.52)	882 (46.13, 2.49)
Gargling with Isozine	8,477 (13.11, 97.28)	102 (11.78, 1.17)	178 (12.09, 2.04)	43 (10.09, 0.49)	237 (12.40, 2.72)
Regular ventilation	31,096 (48.10, 97.72)	316 (36.49, 0.99)	564 (38.32, 1.77)	155 (36.38, 0.49)	725 (37.92, 2.28)
Maintaining humidity	17,723 (27.42, 97.91)	178 (20.55, 0.98)	282 (19.16, 1.56)	81 (19.01, 0.45)	379 (19.82, 2.09)
A well–balanced diet	29,646 (45.86, 98.19)	212 (24.48, 0.70)	420 (28.53, 1.39)	87 (20.42, 0.29)	545 (28.50, 1.81)
Regular exercise	16,209 (25.07, 98.46)	107 (12.36, 0.65)	188 (12.77, 1.14)	41 (9.62, 0.25)	254 (13.28, 1.54)

Getting plenty of rest	31,418 (48.60, 98.08)	268 (30.95, 0.84)	463 (31.45, 1.45)	115 (27.00, 0.36)	616 (32.22, 1.92)
Telework	2,723 (4.21, 97.49)	29 (3.35, 1.04)	59 (4.01, 2.11)	18 (4.23, 0.64)	70 (3.66, 2.51)
Staggered commuting	3,755 (5.81, 97.20)	52 (6.00, 1.35)	84 (5.71, 2.17)	28 (6.57, 0.72)	108 (5.65, 2.80)
Avoidance of crowds other than staggered commuting	13,983 (21.63, 98.01)	104 (12.01, 0.73)	227 (15.42, 1.59)	47 (11.03, 0.33)	284 (14.85, 1.99)
Staying up-to-date on COVID-19	36,718 (56.80, 97.74)	328 (37.88, 0.87)	655 (44.50, 1.74)	133 (31.22, 0.35)	850 (44.46, 2.26)
Other preventive measures	925 (1.43, 97.88)	NA	19 (1.29, 2.01)	NA	20 (1.05, 2.12)
No preventive measures	325 (0.50, 92.86)	15 (1.73, 4.29)	22 (1.49, 6.29)	12 (2.82, 3.43)	25 (1.31, 7.14)

Shiga, n=14,894					
	No symptom (n=14,610, 98.09%)	(a) Fever ≥37.5℃ (n=124, 0.83%)	(b) Strong feeling of weariness or shortness of breath (n=221, 1.48%)	Both (a) and (b) (n=61, 0.41%)	Either (a) or (b) (n=284, 1.91%)
Age, years					
Mean (SD)	45.54 (13.81)	39.94 (16.24)	39.63 (14.79)	40.95 (17.21)	39.48 (14.88)
Range (Min–Max)	13–88	13–80	13–87	13–78	13–87
13–19	474 (3.24, 95.37)	NA	17 (7.69, 3.42)	NA	23 (8.10, 4.63)
20–29	1,291 (8.84, 96.70)	22 (17.74, 1.65)	31 (14.03, 2.32)	9 (14.75, 0.67)	44 (15.49, 3.30)

30–39	2,975 (20.36, 97.29)	37 (29.84, 1.21)	68 (30.77, 2.22)	22 (36.07, 0.72)	83 (29.23, 2.71)
40–49	4,279 (29.29, 98.44)	24 (19.35, 0.55)	54 (24.43, 1.24)	10 (16.39, 0.23)	68 (23.94, 1.56)
50–59	3,196 (21.88, 98.79)	NA	30 (13.57, 0.93)	NA	39 (13.73, 1.21)
60–69	1,739 (11.90, 99.14)	NA	11 (4.98, 0.63)	NA	15 (5.28, 0.86)
70–79	602 (4.12, 98.53)	8 (6.45, 1.31)	8 (3.62, 1.31)	7 (11.48, 1.15)	9 (3.17, 1.47)
80–89	NA	NA	NA	NA	NA
≥90	NA	NA	NA	NA	NA
Sex					

Female	9,922 (67.91, 98.22)	71 (57.26, 0.70)	141 (63.80, 1.40)	32 (52.46, 0.32)	180 (63.38, 1.78)
Male	4,670 (31.96, 97.82)	53 (42.74, 1.11)	80 (36.20, 1.68)	29 (47.54, 0.61)	104 (36.62, 2.18)
Other	18 (0.12, 100.00)	NA	NA	NA	NA
Pregnant	183 (1.25, 98.92)	NA	NA	NA	NA
Occupation					
Self-employed	1,128 (7.72, 98.26)	12 (9.68, 1.05)	15 (6.79, 1.31)	7 (11.48, 0.61)	20 (7.04, 1.74)
Employees	4,809 (32.92, 97.96)	49 (39.52, 1.00)	77 (34.84, 1.57)	26 (42.62, 0.53)	100 (35.21, 2.04)

Public officials	1,057 (7.23, 98.51)	NA	14 (6.33, 1.30)	NA	16 (5.63, 1.49)
Student	762 (5.22, 96.21)	NA	23 (10.41, 2.90)	NA	30 (10.56, 3.79)
Part–time workers	3,165 (21.66, 98.54)	NA	34 (15.38, 1.06)	NA	47 (16.55, 1.46)
Unemployed	2,645 (18.10, 98.18)	22 (17.74, 0.82)	37 (16.74, 1.37)	10 (16.39, 0.37)	49 (17.25, 1.82)
Others	1,044 (7.15, 97.94)	NA	21 (9.50, 1.97)	NA	22 (7.75, 2.06)
Taking antifebrile medications (Loxonin, Caronal, etc.)					

Current	465 (3.18, 84.09)	45 (36.29, 8.14)	69 (31.22, 12.48)	26 (42.62, 4.70)	88 (30.99, 15.91)
Past one month	1,133 (7.75, 95.05)	17 (13.71, 1.43)	53 (23.98, 4.45)	11 (18.03, 0.92)	59 (20.77, 4.95)
Diseases currently undergoing treatment (multiple answers)					
Malignant tumor with anticancer drugs	NA	NA	NA	NA	NA
Malignant tumor without anticancer drugs	NA	NA	NA	NA	NA
Cardiovascular diseases	363 (2.48, 97.06)	NA	10 (4.52, 2.67)	NA	11 (3.87, 2.94)
Kidney diseases	140 (0.96, 95.89)	NA	NA	NA	6 (2.11, 4.11)

Diabetes mellitus	575 (3.94, 97.13)	8 (6.45, 1.35)	16 (7.24, 2.70)	7 (11.48, 1.18)	17 (5.99, 2.87)
In dialysis treatment	NA	NA	NA	NA	NA
Chronic obstructive pulmonary disease	NA	NA	NA	NA	NA
Treatment with immunosuppressant	160 (1.10, 93.57)	8 (6.45, 4.68)	NA	NA	11 (3.87, 6.43)
Preventive measures (multiple answers)					
Washing hands in running water	8,156 (55.82, 97.92)	65 (52.42, 0.78)	139 (62.90, 1.67)	31 (50.82, 0.37)	173 (60.92, 2.08)
Washing hands with soap and water	12,655 (86.62, 98.22)	103 (83.06, 0.80)	173 (78.28, 1.34)	47 (77.05, 0.36)	229 (80.63, 1.78)
Hand disinfection with alcohol	9,352 (64.01, 98.13)	77 (62.10, 0.81)	137 (61.99, 1.44)	36 (59.02, 0.38)	178 (62.68, 1.87)

Etiquette (masks, handkerchiefs, etc.) in case of coughing or sneezing	12,802 (87.62, 98.27)	94 (75.81, 0.72)	173 (78.28, 1.33)	41 (67.21, 0.31)	226 (79.58, 1.73)
Take time off from school or work when you have a fever or other symptoms	5,777 (39.54, 98.10)	58 (46.77, 0.98)	82 (37.10, 1.39)	28 (45.90, 0.48)	112 (39.44, 1.90)
Gargling with water	7,494 (51.29, 98.42)	57 (45.97, 0.75)	88 (39.82, 1.16)	25 (40.98, 0.33)	120 (42.25, 1.58)
Gargling with Isozine	1,942 (13.29, 97.44)	20 (16.13, 1.00)	39 (17.65, 1.96)	8 (13.11, 0.40)	51 (17.96, 2.56)
Regular ventilation	6,395 (43.77, 98.43)	44 (35.48, 0.68)	78 (35.29, 1.20)	20 (32.79, 0.31)	102 (35.92, 1.57)

Maintaining humidity	3,723 (25.48, 98.26)	34 (27.42, 0.90)	47 (21.27, 1.24)	15 (24.59, 0.40)	66 (23.24, 1.74)
A well–balanced diet	6,726 (46.04, 98.78)	44 (35.48, 0.65)	56 (25.34, 0.82)	17 (27.87, 0.25)	83 (29.23, 1.22)
Regular exercise	3,736 (25.57, 98.91)	26 (20.97, 0.69)	26 (11.76, 0.69)	11 (18.03, 0.29)	41 (14.44, 1.09)
Getting plenty of rest	6,852 (46.90, 98.53)	44 (35.48, 0.63)	83 (37.56, 1.19)	25 (40.98, 0.36)	102 (35.92, 1.47)
Telework	NA	NA	NA	NA	NA
Staggered commuting	565 (3.87, 98.26)	NA	8 (3.62, 1.39)	NA	10 (3.52, 1.74)
Avoidance of crowds other than staggered commuting	2,794 (19.12, 98.62)	NA	29 (13.12, 1.02)	NA	39 (13.73, 1.38)

Staying up-to-date on COVID-19	8,264 (56.56, 98.39)	52 (41.94, 0.62)	105 (47.51, 1.25)	22 (36.07, 0.26)	135 (47.54, 1.61)
Other preventive measures	179 (1.23, 96.24)	NA	6 (2.71, 3.23)	NA	7 (2.46, 3.76)
No preventive measures	69 (0.47, 87.34)	NA	8 (3.62, 10.13)	NA	10 (3.52, 12.66)

SD, standard deviation.

* For the purpose of anonymization, all cells with less than five people are indicated as NA. In addition, when the number of persons of cells less than five can be uniquely calculated by a combination of numbers (n, %) of a plurality of cells, all the cells of the combination are set to NA (cells with fewer samples are labeled NA preferentially).
